# Dynamic changes in global microRNAome and transcriptome reveal complex miRNA-mRNA regulated host response to Japanese Encephalitis Virus in microglial cells

**DOI:** 10.1038/srep20263

**Published:** 2016-02-03

**Authors:** Bharti Kumari, Pratistha Jain, Shaoli Das, Suman Ghosal, Bibhabasu Hazra, Ashish Chandra Trivedi, Anirban Basu, Jayprokas Chakrabarti, Sudhanshu Vrati, Arup Banerjee

**Affiliations:** 1 Vaccine and Infectious Disease Research Center, Translational Health Science and Technology Institute, Faridabad, India; 2 Computational Biology Group, Indian Association for the Cultivation of Science, Kolkata, India; 3 National Brain Research Centre, Manesar, Haryana, India; 4 Innovative Life Discoveries Pvt. Ltd., Manesar, Haryana, India

## Abstract

Microglia cells in the brain play essential role during Japanese Encephalitis Virus (JEV) infection and may lead to change in microRNA (miRNA) and mRNA profile. These changes may together control disease outcome. Using Affymetrix microarray platform, we profiled cellular miRNA and mRNA expression at multiple time points during viral infection in human microglial (CHME3) cells. *In silico* analysis of microarray data revealed a phased pattern of miRNAs expression, associated with JEV replication and provided unique signatures of infection. Target prediction and pathway enrichment analysis identified anti correlation between differentially expressed miRNA and the gene expression at multiple time point which ultimately affected diverse signaling pathways including Notch signaling pathways in microglia. Activation of Notch pathway during JEV infection was demonstrated *in vitro* and *in vivo*. The expression of a subset of miRNAs that target multiple genes in Notch signaling pathways were suppressed and their overexpression could affect JEV induced immune response. Further analysis provided evidence for the possible presence of cellular competing endogenous RNA (ceRNA) associated with innate immune response. Collectively, our data provide a uniquely comprehensive view of the changes in the host miRNAs induced by JEV during cellular infection and identify Notch pathway in modulating microglia mediated inflammation.

Japanese encephalitis (JE) is an acute central nervous system inflammatory disease caused by infection with Japanese encephalitis virus (JEV), a small, enveloped, plus-strand RNA virus belonging to the family *Flaviviridae*. About 3 billion people in South-East Asia including India and China are at risk of contracting the disease, however, its pathogenesis remains poorly understood. Thus, there is an urgent requirement for different approaches to combat JE induced pathogenesis.

MicroRNAs (miRNAs) have been emerged as a powerful tool to regulate gene expression through the RNA interference pathway. They are highly conserved, endogenous, small noncoding RNAs. The human genome encodes more than 1,500 miRNAs (miRBase v.17) and forms a complex network that is predicted to regulate more than 30% of protein coding genes^1^,^2^. Based on the 2–7 nt seed sequence match, single miRNA can bind and regulate multiple mRNAs function. Alternatively, a single mRNA can be targeted by multiple miRNAs. Thus, by modulating miRNA abundance, it is possible to fine-tune the expression and function of proteins within the cell in a very precise manner^3^. In context to viral infections, deregulated miRNA can influence disease progression and outcome^4^,^5^. MiRNAs also play a crucial role in the regulation of immune response, by controlling the release of inflammatory mediator that ultimately^6^,^7^ have a significant impact on the magnitude of the inflammatory response^8^,^9^.

In addition to their regulatory roles in diverse biological pathways, cellular miRNAs play vital roles in virus–host interactions. Viral infections to the host are an active dynamics process. Several animal viruses have been demonstrated to cause dramatic changes in cellular miRNAs expression^10^,^11^,^12^ and influence the ability of a virus to replicate or spread^10^,^13^. Therefore, simultaneous identification of miRNA-mRNA through profiling will provide a comprehensive view on host viral interaction and allow us to identify key factors associated with pathogenesis. In the previous studies where miRNA profile has been applied to virus infected cells, emphasize mostly was given on host miRNAs interaction at single time point, providing a static snapshot of infection. In contrast to other Flavivirus, the available research to date provides limited information on the role of host miRNAs play during JEV infection. Few reports are available which are mainly focused only on specific miRNA and its role in JEV induced Neuroinflammation^14^,^15^,^16^,^17^. In context to JEV infection, mRNA microarray profiling or miRNA profiling was done mostly either in infected mice brain or in swine testis cells infected with JEV^18^,^19^,^20^. However, until now there is no comprehensive study reported integrating miRNA and mRNA data obtained from cells of human origin. Since, human is the ultimate dead end host, studying miRNA and mRNA changes during JEV infection will provide better understanding about disease pathogenesis.

In the present study, our aim is to elucidate how JEV infection perturbs host’s miRNA expression and identify signature miRNAs regulated pathways that play pivotal role of host JEV interaction. Using an Affymetrix microarray platform, we have profiled global cellular miRNA and mRNA expression in human microglial (CHME3) cells infected with JEV at multiple time points (6, 24, and 48 h). These time points have afforded us a view of the miRNAs and transcriptomic changes occurring both before and during viral replication. Compared to more traditional microarray platforms, the Affymetrix platform combines customizable arrays on a single chip, with each array containing probes for human pre-miRNA, mature miRNA, small nucleolar RNA (snoRNA) and small Cajal body specific RNA (scaRNA) as annotated in the Sanger miRBase 17. It can discriminate between closely related members of the same miRNA family with significantly less cross-hybridization than other traditional microarray platforms. Further, we have performed quantitative reverse transcription-PCR (qRT-PCR) to validate the miRNA chip results. Using target prediction and pathway enrichment analysis, we have identified key cellular pathways associated with the differentially expressed miRNAs (DEMs) and inversely correlated mRNAs during JEV infection. Finally, we have provided evidence suggesting that JEV infection induces NOTCH signaling pathway activation, down regulating several miRNAs that target multiple genes in NOTCH pathway. Over expression of these down-regulated miRNAs have attenuated JEV induced pro-inflammatory cytokine production.

## Results

### Time specific cellular miRNAs signatures in response to JEV infection in human microglial (CHME3) cells

**Figure 1:**
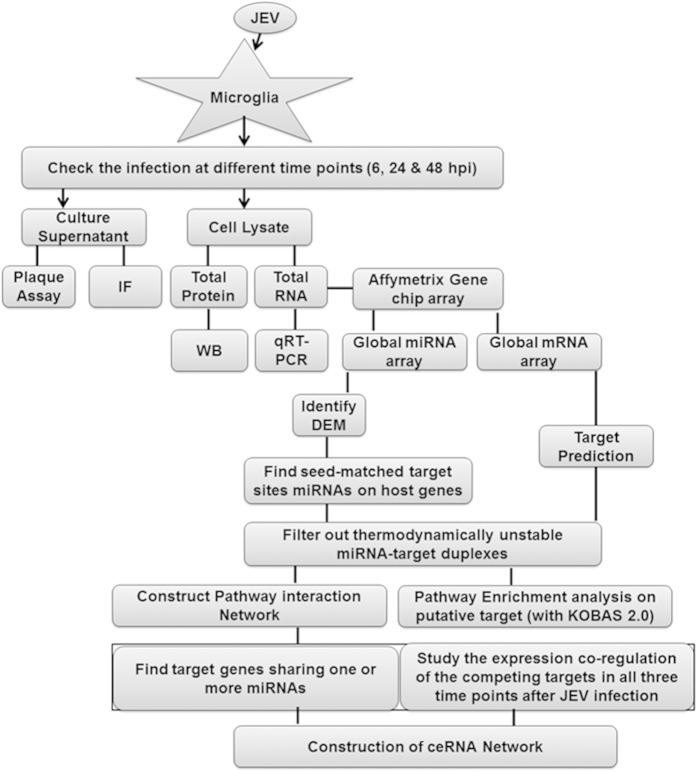
Schematic representation of mRNA and miRNA analysis workflow.

To characterize and measure changes in transcriptome of cells infected with JEV, we seeded *human microglial cells* in six-well tissue culture plates at a density of 0.5 × 10^6^ cells per well and incubated them for 6, 24 and 48 hours post infection (h pi). Cells were infected at high multiplicity of infection (MOI = 5) to enhance infection probability and improve signal to noise ratio and then infected cells were washed with 1× PBS after virus adsorption. We mock-infected a similar number of cells and used them as controls for each time point. The experiment was done in triplicate, Virus infection and replication was monitored at three different time points. The experimental plan and data analysis were outlined in Fig. 1. Viral RNA was detectable as early as at 6 h pi, by qRT-PCR, and the expression level increased at ~500 fold as infection progressed (Fig. S1A, left panel). Increased virus titer was also evident from plaque assay results (Fig. S1A, right panel). Viral NS1protein and envelop protein were detectable only at 24 and 48 h pi checked by western blot (WB) and immunofluorescence (IF) assay as shown in Fig. S1B,C.

**Figure 2:**
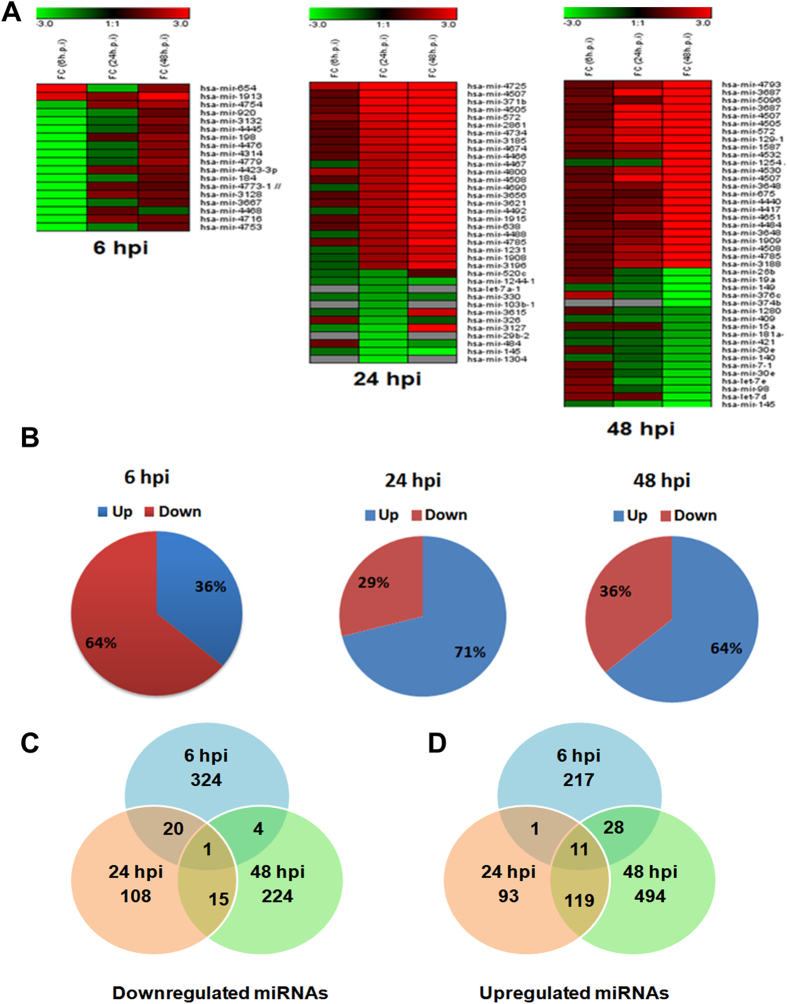
Cellular miRNAs signature in response to Japanese Encephalitis Virus infection in human microglial cell. (**A**) Heat map depicting the snapshot of miRNAs that are differentially expressed at 6 h pi, 24 h pi and 48 h pi. Red denotes upregulation and green denotes downregulation of miRNAs at particular time points. (**B**) Pie diagram are showing percentage of deregulated miRNAs at three different time points. (**C,D**) Venn diagrams show the number of common upregulated and down regulated miRNAs between different time points.

To understand the role of human cellular miRNAs in JEV infection, we profiled the expression of cellular miRNAs following infection with JEV. Cellular miRNA expression was determined using the GeneChip miRNA 3.0 Affymetrix Array Technologies. The expression of highly significantly deregulated miRNA across all time points was depicted with a heat map (Fig. 2A). Fold change of all DEM (FC = 1.5 and P < 0.05) were shown in Table S1. At 6 h pi, 16 of 25 miRNAs demonstrated reduced expression, thereby, displaying the highest number of down regulated miRNAs compared to any other time point. The number of upregulated miRNAs remained relatively low for the first 6 h pi, where only 36% of the total DEM are upregulated, before dramatically increasing at 24 and 48 h pi to 71% and 64% (Fig. 2B). It is important to note that the subset of significantly down regulated miRNAs at early time points during infection is distinct from the subset of significantly upregulated miRNAs at late time points in infection. Only miR-197-3p was found to be down regulated miRNAs, common to all time points (Fig. 2C, Table S2). Eleven upregulated miRNAs were found to be common among each time point (Fig. 2D, Table S2). Several brain enriched miRNAs (miR-128 and miR-132) and other miRNAs (e.g. miR-196, miR-222, and miR-9*, miR-7, miR-130b and miR-126-5p) that were previously shown to be associated with neurodegenerative diseases were downregulated in JEV-infected microglial cells at 48 h pi (Table S3).

### Validation of miRNA expression

**Figure 3:**
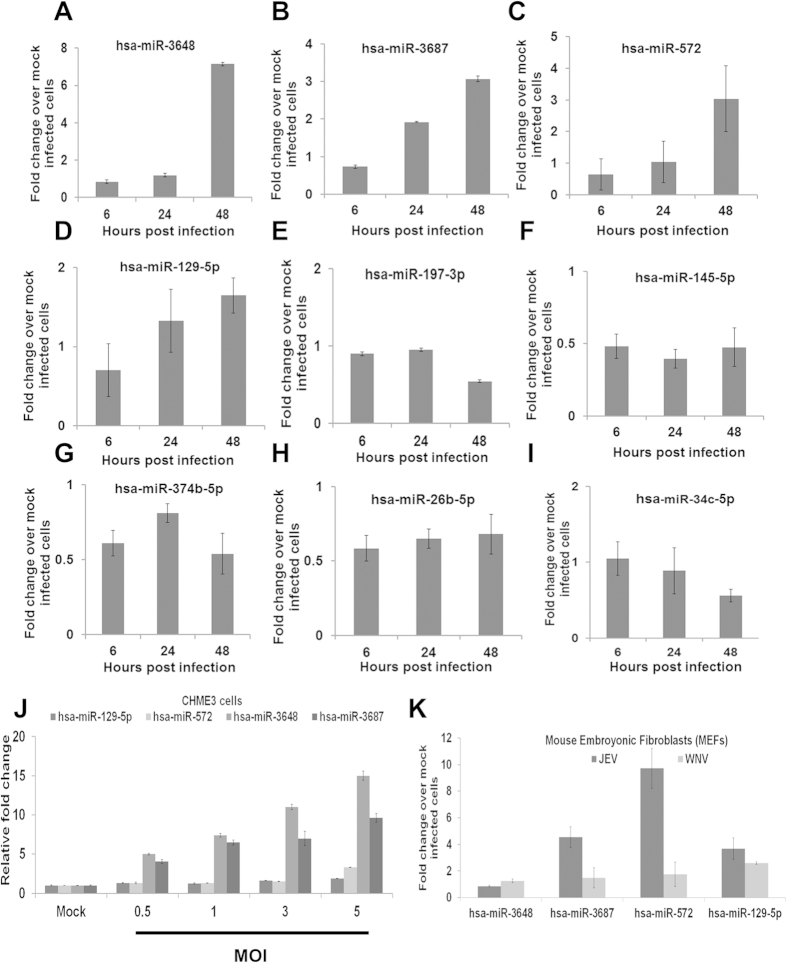
qRT-PCR analysis of miRNA expression of deregulated miRNAs in human microglia cells and Mouse Embryonic Fibroblasts (MEFs) infected with JEV and West Nile Virus (WNV). The results of qPCR analysis of (**A**) miR-3648, (**B**) miR-3687 (**C)** miR-572, (**D**) miR-129-5p, (**E**) miR-197-3p, (**F**) miR-145-5p, (**G**) miR-374b-5p, (**H**) miR-26b-5p, (**I**) miR-149-5p, at three different time points are presented. (J) qRT-PCR graph shows gradual increase in miR-129-5p, miR-572, miR-3648 and miR-3687 expression in CHME3 cells at increasing MOI (0.5, 1, 3 and 5 MOI) after 48 h pi. (**K**) qRT-PCR graph showing miR-129-5p, miR-572, miR-3648 and miR-3687 expression in MEFs cells infected with JEV and WNV after 24 h pi. Each graph represents mean absolute fold change of triplicate experiments for each miRNAs at three individual time point as compared to mock infected controls. All qRT-PCR data are represented as means ± SD. Significance is based on one way ANOVA analysis.

Comparative qRT-PCR analysis was used to further validate the results obtained from our microarray data. Subsets of miRNAs were selected for validation, in particular those significantly deregulated at 48 h pi. Using qRT-PCR miRNA assays, we determined the relative fold change of multiple miRNAs over the course of infection (Fig. 3A–I). Each graph represents the mean absolute fold change of triplicate experiments for each miRNA at each individual time point compared to mock-infected controls collected at each time point. Consistent upregulation across 48 h pi was observed with miR-3648, miR-3687, miR-129-5p, miR-572, and two-way ANOVA confirmed that infection is the main factor in miRNAs deregulation as their expression increased along with increasing viral load (*P* < 0.001) (Fig. 3A–D). This is further supported by the fact that upregulated miRNA expression was dependent on initial viral inoculum used for the infection. As shown in Fig. 3J, the expression of miR-3648, miR-3687, miR-129-5p, miR-572, increased with increased MOI used for initial infection in human microglial cells. Interestingly, the expression of these miRNAs were also enhanced in cells infected with West Nile virus (WNV), but upregulation for miR-3687 and miR-572 were more pronounced and significantly upregulated in JEV infected cells (Fig. 3K). The miRNA miR-197-3p showed down regulation trend during JEV infection across all the time points in this miRNA array experiment, although the qRT-PCR data showed only downward trend for this miRNA at 48 h time points (Fig. 3E).

We have also validated miRNAs that are found to be significantly downregulated at 48 h pi (Fig. 3F–I). The strong correlation in results of qRT-PCR and miRNA array was mutually validating and prompted subsequent data analysis.

### Dynamic change in gene expression profile during JEV infection

**Figure 4:**
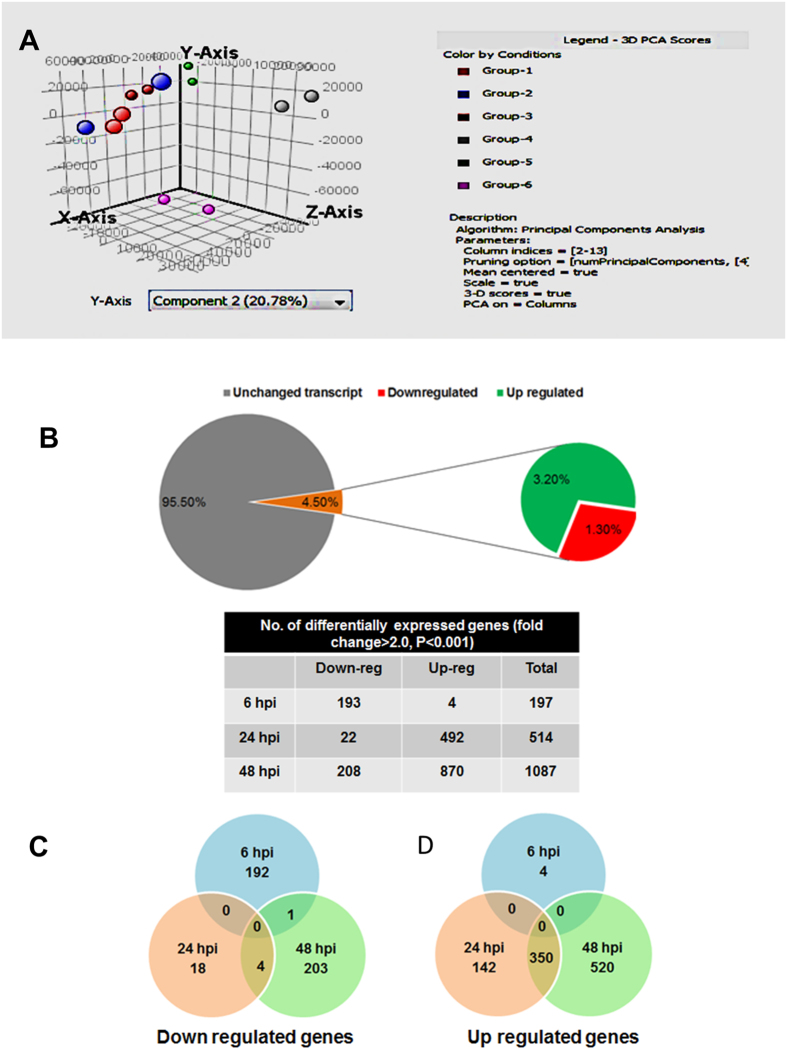
JEV infection in human microglial cells causes changes in cellular genes expression. (**A**) Principal Component Analysis (PCA) of microarray data. The blue, red pink, green, grey, purple circle dots represent liner combination of expression data including relative expression value and variance genes from control and infected duplicate sets of three different time points. (Group -1 = Control 6 h; Group-2 = JEV infected 6 h pi; Group-3 = Control 24 h, Group-4 = JEV infected 24 h pi; Group-5 = control 48 h, Group -6 = JEV infected 48 h pi) (**B**) Using one-way ANOVA, a total of 1434 genes exhibited reproducible change (P < 0.05). This represented 4.5% of the total 32000 transcript on the Affymetrix gene chip. About two-third of deregulated genes were upregulated and rest were downregulated. Table showing the number of deregulated genes expressed following JEV infection at 6, 24 and 48 h pi. (**C**) Venn diagram showing the overlap of differentially expressed (upregulated or down regulated) genes following virus infection.

**Figure 5:**
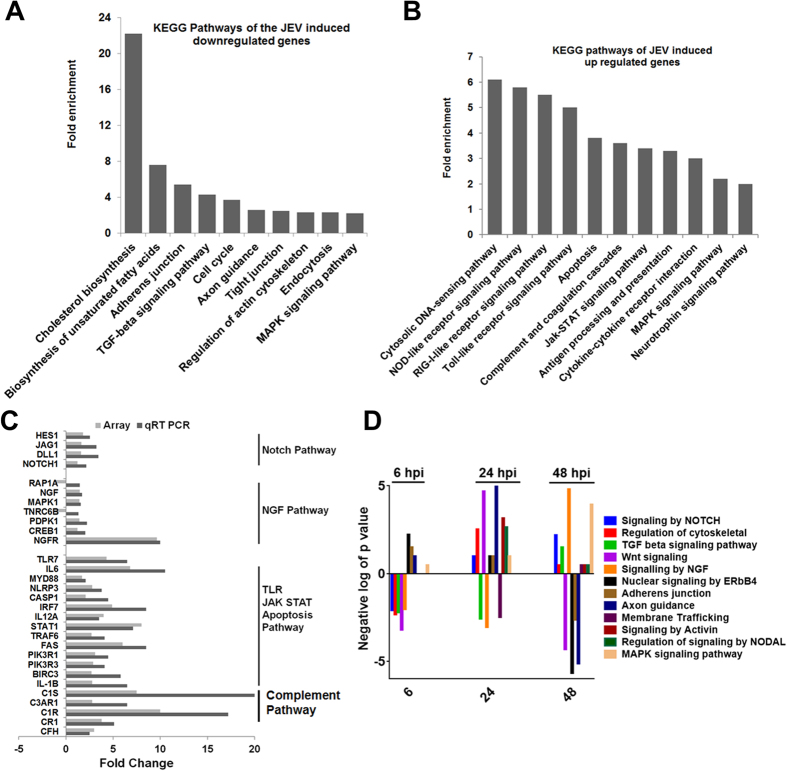
Pathways identified in human microglial cells that are enriched during JEV infection. (**A**) Downregulated or (**B**) upregulated genes were subjected for pathway enrichment analysis using DAVID. Pathway having Fold enrichment score > 2 are depicted in Y axis and pathways are represented in X axis. (**C**) qRT-PCR confirms differential gene expression identified by microarray. qRT-PCR confirmation of selected genes associated with Notch signaling, Neuotrophin signaling, TLR, JAK-STAT, apoptosis and complement pathway. The graphs show changes in expression (infected vs mock) following infection with JEV at 48 h pi, as determined by qRT-PCR and microarray analysis. (**D**) Pathways affected by deregulated miRNAs involved in mRNA-miRNA interaction, are clustered according to pathway ontology categories. Bars bellow the zero line show pathways represented by down regulated mRNAs. On the other hand, bars above the zero line depicts pathway where majority of mRNAs are upregulated at the given time points. The y axis indicates the significance of overlap in the form of negative p values. The X axis represents the hours post infection.

To gain an overall view in the gene expression pattern that may be associated with specific miRNAs expression, we have also performed global mRNA array in CHME3 cells upon JEV infection with similar parameters as for miRNA array. Affymetrix platform and PrimeView GeneChip array type were used to compare gene expression changes in the human microglial cells following JEV infection at multiple time points. Principal component analysis (PCA) indicated that mock infected and JEV infected microglia exhibited divergent gene expression profile and as time progressed, differences in gene expression profile between two groups become more prominent (Fig. 4A). In this study, we identified 1,434 transcripts that exhibited statistically significant changes with P values < 0.05. This represents about 4.5% of the total interrogated host transcripts in the microarray (Fig. 4B). As compared to 24 h pi, a large numbers of genes were found to be significantly down regulated at 48 h pi (Fig. 4C). Interestingly, among the upregulated genes, 350 genes were found to be common between 24 and 48 h pi (Fig. 4D). Therefore, in order to identify the functions of these deregulated genes in context to viral infection in human microglial cells bioinformatics studies were employed. The differentially expressed genes at 24 and 48 h pi time points categorized into diverse functional categories including enzymes, transcription regulators, kinases, nucleic acid binding, transmembrane receptors, transporters, signal transducers and transferases activity (Table S4). To reduce noise in functional analysis and to determine the pathways associated with modulated genes, we applied second filter in data and included genes that exhibited ≥ 2 FC along with the t-test filter with P values < 0.001. Results were scored based on Fisher’s exact test, and functions with P-values ≤ 0.05 were considered statistically significant. Pathways relating to cell signaling, cell cycle, Cholesterol metabolism, amino acid metabolism, axon guidance, tight junction, endocytosis, TGF-beta and MAPK pathway were more significantly altered due to down regulation of genes upon JEV infection (Fig. 5A). On the other hand, upregulated genes were highly enriched in cytokine receptor signaling pathway, Apoptosis, TLR, JAK STAT pathway, complement & coagulation pathways and neurotrophin signaling pathways in human microglial cells (Fig. 5B).

Further, we employed qRT-PCR to validate microarray results. Expression levels of thirty genes involved in five different pathways were assessed and the resulting fold change for each gene was compared with corresponding calculated measurements from the microarray study. The qRT-PCR measurement were carried out in triplicate, and cycle threshold (Ct) values in each observation were normalized to GAPDH to the expression level of the same gene in control mock-infected cells as described in microarray experiments. Despite consistent mild lower fold change values in few assessed genes by qRT-PCR, a direct correlation was evident between the results obtained in both techniques, and 28 out of the 30 gene expression measurements in 48 h pi were concordant (Fig. 5C). Likewise, the observed lower and consistent fold change estimates from qRT-PCR were mainly due to different normalization methods used for both approach. The strong correlation in results of qRT-PCR and microarray was mutually validating and instigated subsequent data analysis.

### Combined miRNA and mRNA microarray data analysis identifies canonical pathways relevant to JEV infection

In order to generate miRNA-mRNA interactome map, we have examined those miRNAs which satisfy the following criteria: a) differentially expressed at three different time points; b) their predicted targets are differentially expressed in gene expression profile; c) the direction of their regulation is antagonistic. The collection of miRNA-mRNA sequences was analyzed using Miranda algorithm to identify potential interaction between them. Using these criteria, we first constructed host miRNA interactome networks for mostly deregulated miRNAs following JEV infection at 6, 24 and 48 h pi. In order to identify the transcripts that might be targeted by DEM in infected microglia, we used the published protocol as described earlier^21^. To identify the biological pathways that become relevant in the human microglia in response to JEV infection, we mapped the target genes of DEM to canonical signaling pathways in the Kyoto Encyclopedia of Genes and Genomes (KEGG). The results showed that 20 statistically remarkable categories (*P* < 0.05) were enriched (Table S5). As shown in Fig. 5D, Neurotrophin signaling pathway (NGF), axon guidance, ErbB pathway, TGF beta signaling pathway, wnt signaling and MAPK kinase pathway are highly enriched during the course of JEV infection. It is evident from the Fig. 5D that the genes involved in Wnt signaling, ErbB4 pathway, Axon Guidance, Adherence junctions were significantly down-regulated and were targeted mostly by up-regulated miRNAs at 48 h pi. On the other hand, signaling by NGF, MAPK, and Notch pathway targeted genes were upregulated at 48 h pi. Table S6 summarizes the list of potential miRNAs and their target genes involved in these different pathways.

### Regulation of the miRNA-target network associated with innate immune response in host microglia

Further, we specifically looked into the potential interaction of deregulated miRNAs with host innate immune response associated genes. The miRNA-target interaction networks for upregulated and downregulated miRNAs at three time points were subjected to pathway enrichment analysis using DAVID^22^ and KOBAS2.0^23^. The predicted target mRNAs from interaction networks were explored further using InnateDB^24^ to identify pathways related to innate immune response that are enriched in network and may have relevance in JEV infection.

From a list of 1535 innate immune response associated genes collected from Innate DB^24^, we found potential interaction of 46 genes with 54 miRNAs deregulated at 48 h pi. Some of these targets shared one or more miRNAs among themselves and showed similar pattern of regulation at all three time points following JEV infection, showing implications of having potential competing endogenous RNA (ceRNA) function^25^,^26^,^27^. Table 1 shows the potential ceRNA pairs associated with innate immune response.

**Figure 6:**
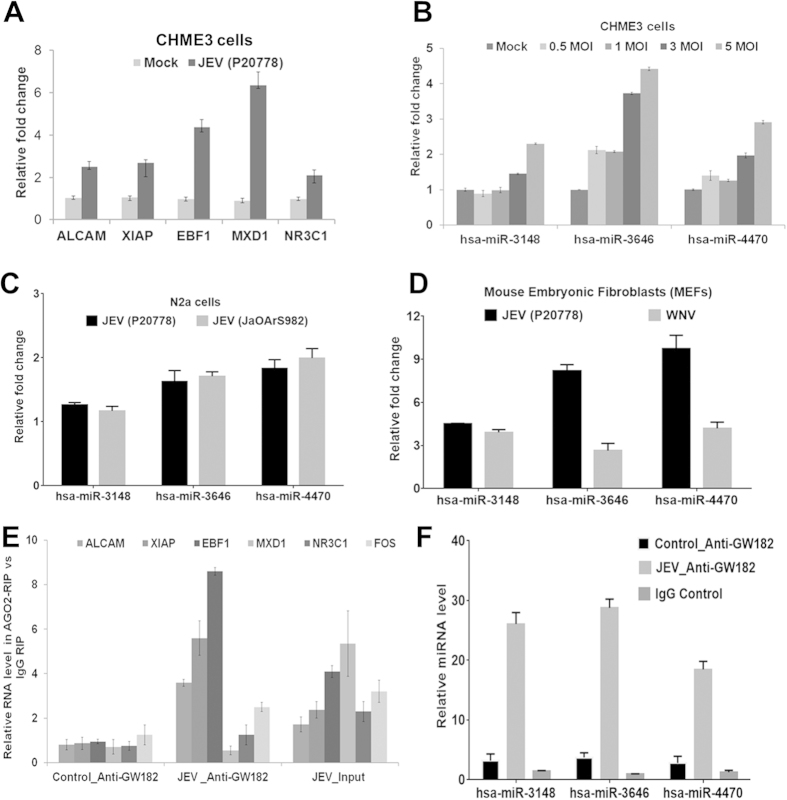
Potential ceRNA network associated genes and microRNAs in JEV infected cells. (**A**) The qRT-PCR graph shows ceRNA genes expression in JEV (P20778) infected CHME3 cells (**B**) The CHME3 cells infected with JEV with different MOI for 48 h and relative miRNA (miR-3148, miR-3646 and miR-4470) expression was measured by qRT-PCR. (**C**) The qRT-PCR graph of these miRNAs expression in JEV (P20778 & JaOArS982) infected N2a cells at 48 h pi. (**D**) Mouse Embryonic Fibroblasts (MEFs) were infected with either with JEV (P20778) or with WNV after 24 h pi and miRNAs were measured by qRT-PCR. (**E**) RIP with mouse monoclonal anti-Ago2, IgG or 10% input from CHME3 cell extracts either mock or infected with JEV for 48 h. RNA levels in immunoprecipitates were determined by qRT-PCR. Levels of ALCAM, XIAP, EBF1, MXD1 and FOS RNA were presented as fold enrichment in Ago2 relative to IgG immunoprecipitates and uninfected control. (F) Relative miRNA levels of miR-3148, miR-4470 and miR-3646 relative to IgG immunoprecipitates and uninfected control. Numbers are mean ± S.D (n = 3).

X-linked Inhibitor of Apoptosis Protein (XIAP), also known as Inhibitor of Apoptosis Protein 3 (IAP3), MAX Dimerization protein 1 (MXD1), A MAPK pathway regulator, *activated Leukocyte Cell-Adhesion Molecule* (*ALCAM*), Early B-Cell Factor 1 (EBF1) and Nuclear Receptor Subfamily 3, Group C, Member 1 (Glucocorticoid Receptor) (NR3C1) were upregulated upon JEV infection at 48 h pi in human microglia and could be targeted by multiple miRNAs (Fig. 6A). Among the several miRNAs that were associated with ceRNA network, we selected only three potential miRNAs (miR-3148, miR-3646 and miR-4470) for validation because these miRNAs can target multiple genes than other miRNAs in ceRNA network. Interestingly, these miRNAs were also upregulated in microglial cells following JEV infection with different MOI, suggesting a positive correlation between viral load and miRNA expression (Fig. 6B). These miRNAs were also overexpressed in neuronal cells (although at lesser extent as compared to microglial cells) and no difference was observed in expression pattern when the cells were infected individually with two different JEV strain (Fig. 6C). These miRNAs were also overexpressed in cells infected with WNV, but significantly higher expression level was observed in JEV infected cells as compared to WNV infection (Fig. 6D).

### ceRNA and miRNAs both co-immunoprecipitates with AGO2 in human microglial cells

MiRNAs are known to be present in the cytoplasm in the form of miRNA ribonucleoprotein complexes (miRNPs) that also contain Ago2, the core component of the RNA-induced silencing complex (RISC). To test whether ceRNA in the network associates with miRNPs, RNA binding protein immunoprecipitation (RIP) experiments were performed on CHME3 cell extracts using antibodies against Ago2. RNA levels in immunoprecipitates were determined by qRT-PCR. XIAP, EBF1 and ALCAM were preferentially enriched (3-8 fold) in Ago2-containing miRNPs in JEV infected cells relative to control immunoprecipitates (Fig. 6E). Similarly, miR-4470, miR-3148, miR-3646 were detected at a level 25–40 fold greater than that of control (Fig. 6D). Successful immunoprecipitation of Ago2-associated RNA was verified by qRT-PCR, using RIP primers against human FOS included in the RIPAb + Ago2 kit (Fig. 6F). Moreover, anti-SNRNP70 was used as a positive control for the RIP procedure, and U1 snRNA was also detected at significant level than that of anti-IgG. Thus, ceRNAs are present in Ago2-containing miRNPs, likely through association with miRNA, consistent with our bioinformatics analysis.

### Reduced expression of microRNAs that target NOTCH signaling pathway during JEV infection

**Figure 7:**
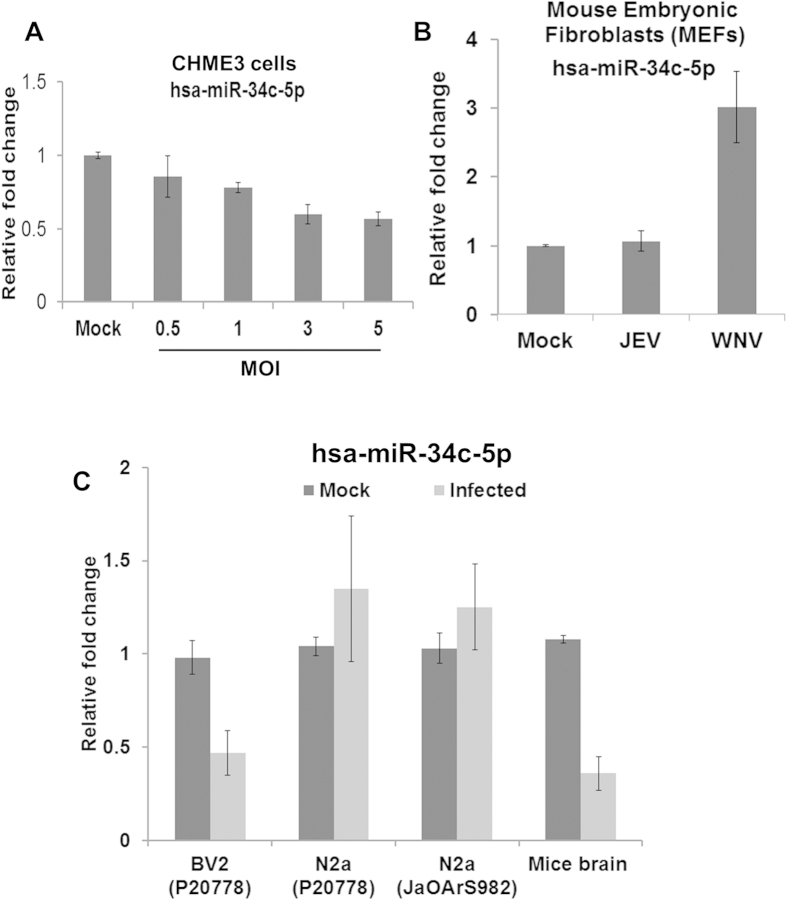
JEV infection specifically suppressed hsa-miR-34c-5p expression in microglial cells. (**A**) Reduced expression of miR-34c-5p was observed in CHME3 cells infected with increasing MOI. The CHME3 cells were infected with JEV (P20778) with different MOI (0.5, 1, 3 and 5 MOI) for 48 h pi and miR-34c-5p expression was measured by qRT-PCR. (**B**) The qRT-PCR graph showing miR-34c-5p expression in MEFs cells after 24 h pi infected with JEV (P20778) and WNV virus. (**C**) Relative miR-34c-5p expression as measured by qRT-PCR in different cell line and mice brain was depicted. All qRT-PCR data are represented as means ± SD. Each graph represents mean absolute fold change of triplicate experiments for miR-34c-5p at particular time point as compared to mock infected controls.

Using bioinformatics analysis, we have found that NOTCH pathway related genes are enriched during JEV infection. Further, miRNA-mRNA interaction study identifies several miRNAs that can target multiple genes in Notch signaling pathway. Table S6 showed the list of miRNAs and their target genes in Notch signaling pathway. In order to understand their role in Notch pathway during JEV infection, we have first validated their relative expression by qRT-PCR. Expression of all miRNAs (miR-145-5p, miR-26b-5p, miR-34c-5p, and miR-374b-5p) was suppressed in varying degree with JEV infection (Fig. 3F–I). Further, we took miR-34c-5p as it can target multiple gene (Notch1, Dll1, JAG1, Hes1) expression in Notch signaling pathway. To understand if the miR-34c suppression is JEV specific, we infected the microglia cells with JEV having varying MOI and miR-34c-5p expression was examined after 48 h pi. A gradual repression of miR-34c-5p expression was observed with increasing MOI (Fig. 7A). To check further if this suppression is related to JEV infection, we compared miR-34c-5p expression in WNV infected cells. WNV infection induced miR-34c -5p upregulation was observed in mouse embryonic Fibroblast cells, but no change was noted in MEFs infected with JEV (Fig. 7B). No significant change was observed in miR-34c-5p expression in neuronal cells infected with two different strains, but significant suppression was observed in mouse microglia as well as mouse brain infected with JEV (Fig. 7C). These results further instigated us to check Notch signaling pathway during JEV infection in context to microglia cells.

### Notch pathway is activated during JEV infection

**Figure 8:**
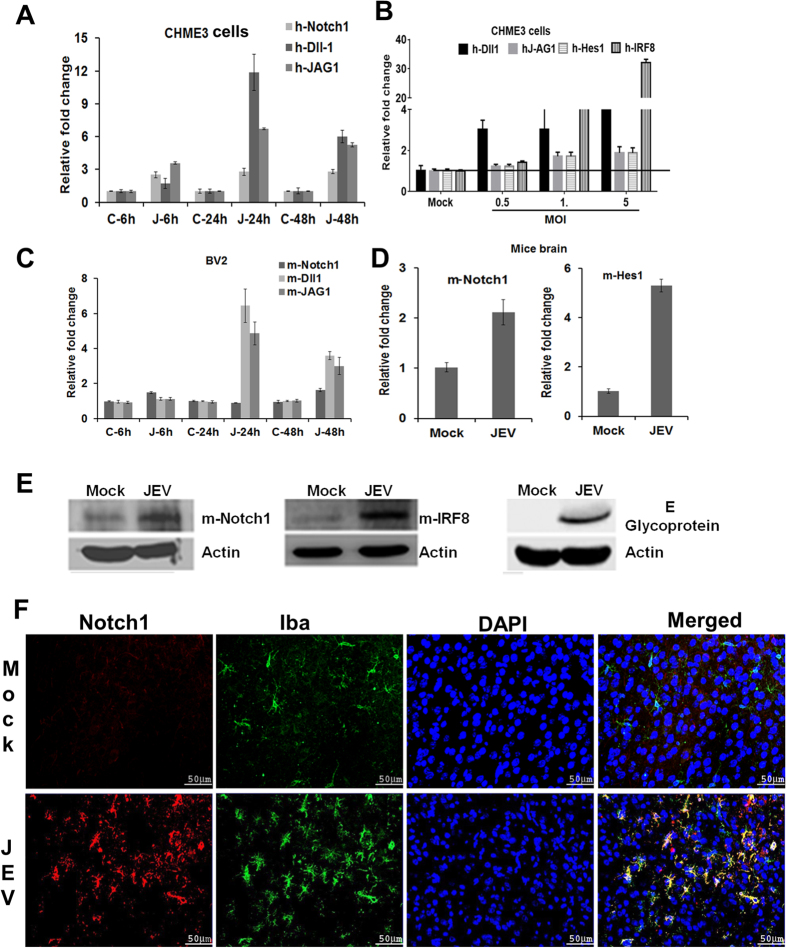
NOTCH pathway is activated during JEV infection. (**A**) Validation of Notch1 receptor, ligands Dll1 and JAG1 expression in CHME3 cells infected with JEV (P20778) at three different time points by qRT-PCR. (**B**) The qRT-PCR graph showing ligands Dll1, JAG1 and their target genes Hes1 and IRF8 expression in JEV (P20778) infected CHME3 cells at different MOI (0.5, 1 and 5 MOI) after 48 h pi. (**C**) Confirms Notch1, Dll1 and JAG1 expression in JEV (P20778) infected BV2 cells at different time points by qRT-PCR. (**D**) Validate Notch1 and Hes1 expression in JEV infected mice brain by qRT-PCR. All qRT-PCR data are represented as means ± SD. Each graph represents mean absolute fold change of triplicate experiments for each mRNAs at three individual time point as compared to mock infected controls. (**E**) Western blot analysis of activated Notch1, IRF8 and JEV envelope protein are shown in infected mice and mock brain lysate. Mouse Actin is used as internal control. (**F**) Immunohistochemistry was done on infected mice or Mock brain section. Activated microglia is stained with Iba (green), activated Notch (NICD) is stained with red and DAPI is used to stain nucleus.

In the central nervous system (CNS), the Notch signaling pathway play important role in microglia activation. Mammalian canonical Notch signaling is activated when either the Delta or the Jagged ligands bind to one of four Notch receptors, thus resulting in a two-step proteolytic cleavage which ultimately releases the Notch intracellular domain (NICD) from the membrane^28^. Hes1 is a transcription factor, whose expression is initiated by the Notch signaling pathway^28^. Transcription of IRF8 is directly regulated by Notch pathway. Our mRNA array data suggested an up-regulation of Notch1, Dll1, JAG1 and Hes1 expression upon JEV infection which was further validated by qRT-PCR at three different time points (Fig. 8A). Notch ligand and target genes expression was further upregulated in CHME3 cells infected with different MOI (Fig. 8B). Notch mRNA expression was also upregulated in BV2 and mice infected brain tissues (Fig. 8C,D). Activated Notch expression was also elevated in infected mice brain as shown in Fig. 8E. Transcription of IRF8 is directly regulated by Notch pathway. We have previously reported that IRF8 is overexpressed during JEV infection in CHME3 cells^14^. Here, we further showed that IRF8 expression was also increased in JEV infected mice brain. We further examined activated Notch (NICD) expression in control and JEV infected mice brain. Immunofluorescence images showing NICD expression in activated microglia labeled with Iba; (green). The expression is intensely augmented both in the cytoplasm and nucleus after JEV infection compared with the mock infection (Fig. 8F). Together, these results suggested that Notch pathway is activated during JEV infection.

### Overexpression of microRNAs targeting Notch1 attenuated JEV induced proinflammatory cytokine production

**Figure 9:**
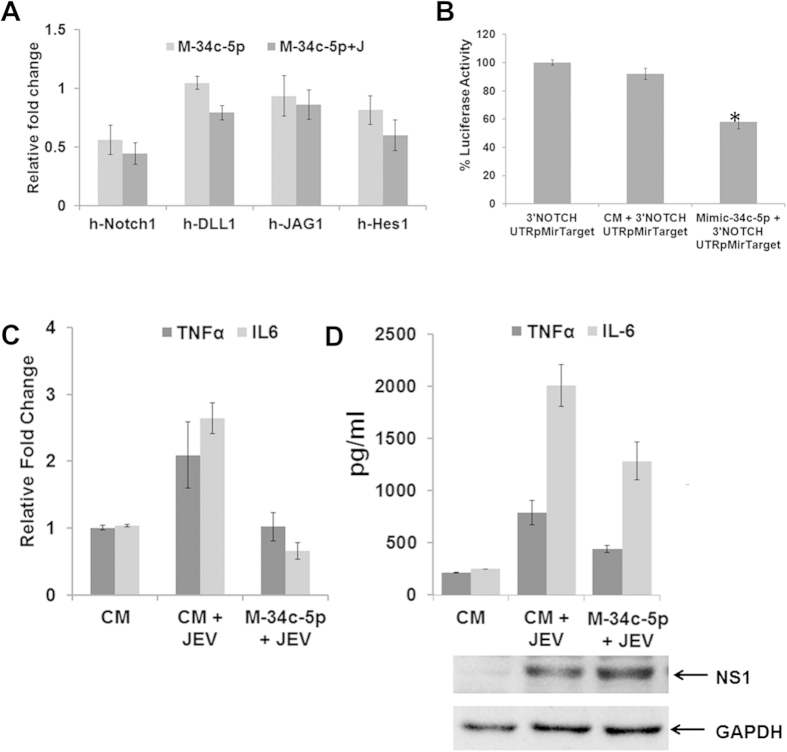
Overexpression of miR-34c-5p attenuates JEV induced proinflammatory cytokine expression. (**A**) miR-34c overexpression in CHME3 cells suppressed JEV induced Notch1, Dll1, JAG1 and Hes1 expression. (**B**) Overexpression of miR-34c probably binds 3’UTR of Notch gene and repressed gene expression. Cells were transfected with pMirTarget Luciferase vector (Origene, Rockville, MD, USA) containing wild type 3’UTR of Notch in the presence of either control mimic (CM or mimic-34c (M-34c) for 48 h before being harvested for analysis. Statistical analysis was calculated using Student t test and expressed as percent of Luciferase activity. (**C,D**) Inflammatory cytokine expression is attenuated upon overexpression of Notch pathway targeted miRNA. Mouse BV2 cells were transfected either with control mimic or with M-34c for 24 h. After that, cells were infected with JEV and cells were lysed after 24 h pi for RNA extraction and cell culture supernatant was subsequently collected for CBA array. (**C**) The graphs depict the mean mRNA expression of TNF-α and IL6 in the respective samples. (**D**) Secreted protein concentration was measured by CBA. JEV protein expression was demonstrated by Western Blot. The GAPDH was used as internal control (Lower panel). The graphs represent the concentration in pg/ml of cytokines TNF-α and IL6. Values represent Mean ± SEM from two independent experiments performed in triplicate.

Recent evidence suggests that Notch1 may also play an important role in regulating the responsiveness of immune cells to stimulation and infection^29^,^30^,^31^,^32^. In order to understand that these miRNAs are really affect Notch1 pathway, we have checked Notch1, Dll1, JAG1 and Hes1 gene expression after overexpressing the miR-34c-5p by transfecting miRNA specific mimic followed by JEV infection. Our results suggested that overexpression of miRNAs led to repression of Notch1 and Hes1 expression in human microglial cells with or without JEV infection (Fig. 9A). Further, we provided experimental evidence suggesting miR-34c-5p probably binds 3’UTR of Notch gene as overexpression of miR-34c-5p repress luciferase activity in the HEK293T cells when co-transfected with Notch 3’UTR pMirTraget Luciferase vector (Fig. 9B). In order to know the effect of Notch1 pathway inhibition on JEV induced inflammatory cytokine production, we overexpressed Notch pathway targeted miRNA and checked mRNA and protein expression of TNFα and IL-6 by qRT- PCR (Fig. 9C) and by CBA (Fig. 9D, upper panel) after JEV infection. Overexpression of hsa-miR-34c-5p, led to attenuation of JEV induced TNF and IL-6 expression, both at mRNA and protein level, but this overexpression has no effect on JEV replication as no change in viral specific NS1 protein expression was observed in miR-34c-5p overexpressed cells as compared to control mimic transfected cells (Fig. 9D, lower panel).

## Discussion

The results of our study provide the first experimental evidence demonstrating the complex temporal regulation of host microRNAome by JEV infection in human microglia cells. The study is unique in many aspects. First, we have used human microglia cells to understand host miRNA regulation by JEV infection. To gain comprehensive information on host miRNA-mRNA interaction upon JEV infection, we have profiled miRNA as well as mRNA at three different time points (6, 24, 48 h). These time points afford us a view of the miRNAs and transcriptomic changes occurring both before and during viral replication. Second, the integration of array chip, qRT-PCR, and target prediction followed by pathway analysis has allowed us to perform a robust comparative genomics and bioinformatics study to reveal host miRNA molecular signature associated with JEV infection. We have identified a unique series of temporal host molecular responses involving different combinatorial contributions of multiple cellular miRNAs. This provides the important molecular understanding about the unique cellular miRNA-mRNA interactome networks, dynamically and temporally regulated by JEV infection. Third, this study also looks into the presence of cellular competing endogenous RNA (ceRNA). These ceRNAs impose another level of regulation in miRNA-mRNA network. Our integrated bioinformatics analysis identifies ceRNAs that may involve in regulating host innate immune response or directly compete with viral genome against the particular miRNA. Finally, we have verified Notch pathway which is enriched in pathway analysis during JEV infection. We have provided experimental evidence suggesting that Notch pathway is activated during infection and is linked to inflammatory cytokine production.

Several studies have demonstrated changes in miRNAs expression level in response to Flaviviral infection. In context to JEV infection, until now there is no comprehensive study reported integrating miRNA and mRNA data obtained from cells of human origin. To our best knowledge, this is the first report where effect of JEV infection on host miRNAs and mRNAs expressions was studied extensively in human microglial cell. To understand the impact of strain and cell type specific variation on DEM expression, we examined seven upregulated and one down regulated miRNAs expression pattern in neuronal cells infected with two different strains. Our results suggested that strain specific variation may be marginal, but cell type specific variation may exist, especially, we observed microglial cell type specific expression pattern for miR-34c-5p (Fig.7c). Although upregulation pattern of seven miRNAs was noted in cells infected with WNV, enhancement is more pronounced in JEV infected cells especially for miR-3646, miR-4470 (Fig. 6C), miR-572 and miR-3687, suggesting their possible JEV specific role during infection. Interestingly, miR-34c-5p highly expressed in WNV infected cells which is totally opposite to the phenomenon that we observed during JEV infection (Fig. 7B). This further suggest that suppression pattern of miR-34c is specific for JEV and might linked to JEV specific pathogenesis.

Recently new classes of regulatory RNA concept have evolved called competing endogenous RNA (ceRNA). The ceRNA which includes both linear and circular RNAs competes with specific mRNAs for providing binding sites to the corresponding miRNAs. A particular ceRNA controls the suppressive effect of a specific miRNA on mRNA translation through sequestering this miRNA, thus facilitating translation of the target mRNA. Further, more recent studies provide convincing evidence that the cross talk between ceRNA through competition for their shared miRNAs is involved in signaling pathways and network in diverse human diseases. Several recent studies also provide intriguing evidence that viral RNA and host miRNAs with common MRE reciprocally affect each other’s level and activity by directly competing with the targeting miRNAs. The ceRNA networks for Hepatitis B, Herpes Simplex virus and Human cytomegalo virus have already been reported^33^. In order to identify if any such ceRNA associated with JEV infection, we first try to identify miRNAs that can target innate immune genes. Using stringent algorithm, we have identified ten miRNAs (miR-129-5p, miR-3148, miR-4470, miR-4672, miR-3646, miR-3180, miR-4690, miR-3622, miR-5096, and miR-885) that can target different genes in innate immune pathway. Using ceRNA identifying algorithm, we finally derive eight mRNA that can act as ceRNAs and share MRE of multiple miRNAs in their 3’UTR. Among them expression of ALCAM, XIAP, MXD1, EBF1and miRNAs was validated and their presence in RISC suggest that they likely interact with miRNAs to control innate immune pathways. The activated leucocyte cell adhesion molecule (ALCAM), also called CD163 plays pivotal in leucocyte transmigration across the blood brain barrier (BBB), especially during neuroinflammatory process^34^. ALCAM is usually expressed on the cells of monocyte-macrophage origin. It is reported that the expression of ALCAM or CD163 on macrophages may be critical in HIV immunopathogenesis^35^. During JEV infection, it is evident that BBB dysfunction is associated with enhanced leucocyte migration in the brain. In this study, using extensive bioinformatics analysis we have identified ALCAM in a ceRNA network and it’s expression is enhanced during JEV infection both in human microglial cells (Fig. 6A) and in mice brain (data not shown). Further, presence of ALCAM in the RISC along with other co-regulated genes as well as miRNAs in ceRNA network provides another level of complexity in innate immune gene regulation during JEV infection. Further study is in progress to understand the role of ceRNAs in JEV pathogenesis.

In order to understand the impact of global miRNA modulation during JEV infection, we further analyzed DEM mediated pathways that have significance in pathogenesis. Our study has provided evidences that JEV induced Notch expression can be regulated through miRNAs and may involve in JEV induced proinflammatory cytokine production. We have shown that Notch pathway is enriched and activated due to JEV infection. Recent evidence suggests that Notch may also play an important role in regulating the responsiveness of immune cells to stimulation and infection^29^,^30^,^31^,^32^. Microglia, one of the most important immune cells in the CNS, contributes in diverse roles within the CNS, and helps to maintain normal brain function. It has only recently been reported that Notch signaling pathway is activated in microglia and plays important role in mediating microglial maturation and activation^32^,^36^,^37^,^38^,^39^. Inhibition of Notch activation by gamma secretase inhibitor reduces proinflammatory cytokine production in activated macrophage^40^. Our study reveal that Notch pathway targeted miRNA (miR-34c-5p) is suppressed during JEV infection and their upregulation impair JEV induced inflammatory cytokine production. Recent report further supported that deletion of Notch 1 in activated macrophages has significant impact on cytokine production and may affect the severity of Experimental Autoimmune Encephalomyelitis (EAE)^41^. Notch activation is also reported from other Flaviviruses. Dengue and Hepatitis C viral NS3 protein can activate Notch pathway^42^,^43^. Right now, we do not know if any JEV viral proteins involve in Notch activation. Since Notch signaling is involved in regulating TLR-mediated responses in activated macrophages and microglia, further study will be needed to underline in depth molecular mechanism.

Overall, our results demonstrate a series of complex sequential host microRNA molecular signatures associated with JEV infection and offers the basis for future investigation. We identify miR-34c-5p which is suppressed during JEV infection and overexpression of this miRNA modulates JEV induced proinflammatory cytokine production possibly through downregulating Notch activation.

## Materials and Methods

### Ethics statement

Animals were handled in strict accordance with good animal practice as defined by the Committee for the Purpose of Control and Supervision of Experiments on Animals (CPCSEA) and the Ministry of Environment and Forestry, Government of India. All animal experiments were approved by the Institutional Animal and Ethics Committee (IAEC) of the National Brain Research Centre. All experiments were performed in accordance with the approved guidelines and regulations.

### Virus infection in cell lines and mice model

The P20778 strain of JEV was propagated in PS cells and titrated by plaque assay^14^. Human microglial cells (CHME3) and mouse microglial cells (BV2) were provided by the National Brain Research Centre, Manesar, India. Porcine stable kidney (PS) cell line, Neuro2a (N2a) cell line were procured from National Centre for Cell Science, Pune, India. The JaOArS982 and West Nile Virus (NY99) (WNV) were propagated in C6/36 insect cells and Vero cells respectively and N2a and MEFs cells were infected with these viruses with the MOI of 5 for 36 h. A previously described animal model of JE^44^ was used for *in vivo* studies.

### Antibodies, miRNA primers and mimics

Primary antibodies against, IRF-8 rabbit polyclonal antibody (1:10,000), anti-activated Notch1 rabit polyclonal antibody (1:500, Abcam, USA) and HRP-conjugated secondary antibodies, anti-rabbit and anti-mouse (1:10,000) were purchased from Cell Signaling Technology (Beverly, MA, USA). Rabbit polyclonal antibody against JEV NS1 protein was produced in-house. GAPDH rabbit polyclonal antibody (1:10,000) was from GeneTex (Irvine, CA, USA). MicroRNA mimics and primers were from Sigma-Aldrich (Saint Louis, MO, USA), negative control mimic, MC10722, mirVana® miRNA Mimic Control #1.

### MicroRNA expression profiling using Affymetrix miRNA 3.0 array: RNA Isolation, quality control and hybridization

Total RNA was isolated from the cell lines using RNeasy Mini kit according to the manufacturer’s instructions (Qiagen, Hilden, Germany). Samples were collected from six-well plate in triplicate for each of the following time points: 6, 24 and 48 hours post infection (h pi) at multiplicity of infection (MOI) 5. Samples were frozen at −80 °C for subsequent use in microarray experiments. RNA integrity number (RIN) was ascertained using Agilent 2100 Bioanalyzer (Agilent Technologies Inc., USA). Fragmentation, hybridization and scanning were performed according to the Affymetrix miRNA protocol, using the miRNA 3.0 array covering 5,339 probes for the human (Affymetrix, Santa Clara, CA, USA) (For details see Supplementary methods).

### Prediction of host and viral genome targets of deregulated miRNAs at different time points

Target genes of the differentially expressed miRNAs (DEM) isolated by profiling were identified by combinatorial computational analysis using multiple web-based prediction algorithms. We collected putative human miRNA targets on human protein coding transcripts from TargetScan^45^. For prediction of deregulated host miRNA targets on JEV genome, we used a custom algorithm developed by our group. This custom algorithm for seed-matched miRNA target finding was used in our previous works^25^,^46^. The custom algorithm used seed matched target searching coupled with favorable duplex stability. For seed complementarity search, a modified version of Smith-Waterman algorithm as used in Miranda miRNA target prediction software^47^ was used and for prediction of different types of miRNA target sites (6-mer, 7-mer, 7-merA1 and 8-mer). We considered transcripts with seed complementarity as well as one base mismatch tolerance (in position 2–8 or 2–7) in the seed region with 3’ compensatory complementarities. The seed-matched potential targets were further subjected to calculation of miRNA-target duplex energy. The predicted miRNA-target duplexes with energy > −20 kcal/mol were filtered out as unfavorable. The algorithm was customized for reducing runtime involved in computation of miRNA target sites in genome-wide scale^46^.

### Pathway enrichment, gene ontology study and construction of miRNA-target interaction network

Pathway and Functional analysis for the differentially regulated genes and the genes showing inverse correlations was done using Database for Annotation, Visualization and Integrated Discovery (DAVID) Gene Ontology tool (http://david.abcc.ncifcrf.gov/) available online with PANTHER and KEGG Pathways as the selected databases. Genes were classified into functional categories according to the Gene Ontology. To identify biological processes most involved in the biological phenomena under study we have performed a Gene Ontology (GO) analysis, using DAVID tool.

The miRNA-mRNA anti-correlations were visualized by Cytoscape software package^48^. The predicted miRNA-target pairs were checked for co-expression in three time points after JEV infection. The co-expressed miRNA-target pairs were used for constructing interaction network in Cytoscape.

### Prediction of potential ceRNA pairs

We implemented a measure to assess the likelihood of a host transcript sharing miRNA targets with JEV to act as a ceRNA. This approach was similar to what has been used in our previous works^25^,^46^ and the study of Sumazin *et al*.^26^ and in StarBase v2.0^49^. We calculated the p-value for each potential ceRNA pair by hypergeometric test considering the number of shared miRNAs between a ceRNA pair against the number of miRNAs targeting individual components of the ceRNA pair. The p-value was measured by the equation 1: 
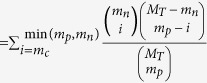
, 23 where, 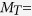 Total number of miRNAs in the human genome; 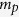 = Number of miRNAs interacting with the mRNA (protein-coding), 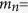 Total number of miRNAs interacting with the lncRNA (non protein-coding), 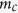 = Number of miRNAs shared between the ceRNA pair.

### RISC IP

RNA immunoprecipitation was performed using the EZ-Magna RIP kit (Millipore, Billerica, MA, USA) following the manufacturer’s protocol. CHME3 cells either infected with JEV for 48 h or Mock at 80–90% confluent were scraped off and then lysed in complete RIP lysis buffer, after which 100 μl of whole cell extract was incubated over night with RIP buffer containing magnetic beads conjugated with anti-Ago2 antibody (Millipore, USA) and negative control normal mouse IgG (Millipore, USA). JEV infection was examined in input samples using specific primer set. Anti-SNRNP70 (Millipore, USA) was used as positive control for the RIP procedure. After 18 hours, samples were incubated with Proteinase K with shaking to digest the protein and then subsequently immunoprecipitates RNA was isolated. The RNA concentration was measured using a NanoDrop (Thermo Scientific, USA). Furthermore, using respective miRNA and gene primers, purified RNA was subjected to qRT-PCR analysis to examine the presence of the binding targets in the RISC complex.

### qRT-PCR

For validation of mature miRNAs expression, 200 ng of total RNA was reverse transcribed *in vitro* to cDNA using the MystiCq microRNA cDNA Synthesis Mix (Sigma-Aldrich, Saint Louis, MO, USA) according to the manufacturer’s instructions. Relative expression was calculated using the Ct method with uninfected as the reference and small nuclear RNA U6 as endogenous controls.

JEV RNA was quantified as described earlier^14^. Similarly, RNA was extracted from JEV infected mice brain and uninfected control brain. Notch1, Dll1, JAG1, Hes1 expression were examined with specific primer by qRT-PCR method. The RNA transcript levels were normalized to that of

### Immunoblotting

Mouse brains were lysed and immunoblotting of isolated protein samples was performed according to a standard procedure as described earlier^15^,^16^. Briefly, equal amounts of protein (50 μg) were separated with 10% SDS-PAGE and transferred onto a nitrocellulose membrane. The membrane was probed with either rabbit activated Notch1 antibody (1:500 dilution) (Abcam, USA), or rabbit IRF8 antibody to check their expression in infected and uninfected mice brain.

### Immunohistochemistry

The immunohistochemical staining of mouse brain sections was carried out as said previously^16^ to study the co-expression of activated Notch1 and microglial Iba protein. After perfusion with ice-cold PBS, brains of sacrificed mouse were excised and fixed with 4% paraformaldehyde. The sections of 20 μm thick were prepared using a Leica CM3050S cryostat and after processing incubated overnight with anti-Notch1 (1:250; Abcam) and anti-Iba (1:250; Millipore) at 4 °C. Next day, after extensive washing, the sections were incubated with Alexa Fluor 488 and Alexa Fluor 594 (1:1,500; Molecular Probes, Eugene, OR) conjugated secondary antibodies. Finally, the sections were mounted with 4′-6-diamidino-2-phenylindole (DAPI; Vector Laboratories Inc., California, USA) and observed under Zeiss Apotome microscope (Zeiss, Gottingen, Germany) at the specified magnification.

### Transfection of cells with mimic, 3’UTR Luciferase vector and virus infection

Microglial cells were seeded in six-well tissue culture plates at a density of 0.5 × 10^6^ cells/well and mimics were transfected 24 h later using Lipofectamine 2000® reagent (Invitrogen, Carlsbad, CA, USA) according to the manufacturer’s protocol. These miRNA mimics were transfected into CHME3 or BV2 cells at a final concentration of 25 μM Cells were washed with 1× PBS after 24 hours transfection and infected with JEV at MOI = 5. Culture supernatant was collected to perform cytokine bead array. The cells were used for RNA studies. Luciferase reporter assay was performed as described earlier^50^. Briefly, Control mimic or mimic-34c-5p and UTR piRMIR Target vector were co transfected and Luciferase readout was measured after 48 h of transfection.

### Cytokine Bead array (CBA)

CBA was performed to quantitatively measure the cytokine level (IL-6, TNFα, IFN-γ, MCP1, IL-12p70, IL-10) in culture media from BV2 cells according to manufacturer protocol and analyzed using CBA software. The quantity of the cytokine released in culture supernatant will be measured against the standard curve obtained from defined concentration of protein.

### Statistical analysis

All experiments were performed in triplicate. Gene expression profiling data were analyzed statistically using one way analysis of variance (ANOVA) following Bonferroni’s multiple comparison tests. Data were presented as the mean ± SD; statistical significance of difference (P value) for two means was assessed using an unpaired Student’s t-test using the GraphPad Prism 5 software (GraphPad, San Diego, CA, USA), and P < 0.05 was considered significant.

### Microarray data resource

The microarray data was deposited in the Gene Expression Omnibus (GEO) database under accession number GSE57647.

## Additional Information

**How to cite this article**: Kumari, B. *et al*. Dynamic changes in global microRNAome and transcriptome reveal complex miRNA-mRNA regulated host response to Japanese Encephalitis Virus in microglial cells. *Sci. Rep*. **6**, 20263; doi: 10.1038/srep20263 (2016).

## Supplementary Material

Supplementary Information

## Figures and Tables

**Table 1:** Predicted ceRNA pairs associated with innate immune response in JEV infected microglia cells.

**ceRNA 1**	**ceRNA 2**	**Number of shared miRNAs**	**Shared miRNAs**
ALCAM	EBF1	2	hsa-miR-3148, hsa-miR-4470,
ALCAM	MXD1	1	hsa-miR-3148,
ALCAM	TCF4	1	hsa-miR-3148,
ALCAM	XIAP	2	hsa-miR-3148, hsa-miR-4470,
EBF1	MXD1	4	hsa-miR-3148, hsa-miR-4672, hsa-miR-3646, hsa-miR-3180-5p,
EBF1	TCF4	4	hsa-miR-3148, hsa-miR-129-5p, hsa-miR-5096, hsa-miR-3646,
EBF1	XIAP	4	hsa-miR-3148, hsa-miR-4470, hsa-miR-5096, hsa-miR-3646,
MXD1	TCF4	3	hsa-miR-3148, hsa-miR-3646, hsa-miR-885-5p,
MXD1	XIAP	3	hsa-miR-3148, hsa-miR-3646, hsa-miR-4690-5p,
TCF4	XIAP	3	hsa-miR-3148, hsa-miR-5096, hsa-miR-3646,
ALCAM	CD2AP	1	hsa-miR-4470,
CD2AP	EBF1	1	hsa-miR-4470,
CD2AP	XIAP	1	hsa-miR-4470,
EBF1	NR3C1	3	hsa-miR-4672, hsa-miR-5096, hsa-miR-3646
MXD1	NR3C1	2	hsa-miR-4672, hsa-miR-3646,
CD2AP	TCF4	1	hsa-miR-3622a-5p,
NR3C1	TCF4	2	hsa-miR-5096, hsa-miR-3646,
NR3C1	XIAP	2	hsa-miR-5096, hsa-miR-3646,
MXD1	UBE2H	1	hsa-miR-885-5p,
TCF4	UBE2H	1	hsa-miR-885-5p,
