# Supplementary material for: Dynamic changes in global microRNAome and transcriptome reveal complex miRNA-mRNA regulated host response to Japanese Encephalitis Virus in microglial cells
Source: Sci Rep. 2016 Feb 3;6:20263. doi: 10.1038/srep20263 (PMC4738309; doi:10.1038/srep20263)
Supplement: Supplementary Information [file srep20263-s1.pdf]

Revision 1 **SREP-15-31644A** **Supplementary Documents**

Dynamic changes in global microRNAome and transcriptome reveal complex miRNA-mRNA regulated host response to Japanese Encephalitis Virus in microglial cells

Running Title: microRNA signature in JEV infected microglia

Bharti Kumari[1], Pratistha Jain[1], Shaoli Das[2], Suman Ghosal[2], Bibhabasu Hazra[3], Ashish Chandra Trivedi[4], Anirban Basu[3], Jayprokas Chakrabarti[2], Sudhanshu Vrati[1], Arup Banerjee[1*]

[1]Vaccine and Infectious Disease Research Center, Translational Health Science and Technology Institute, Faridabad, India

[2]Computational Biology Group, Indian Association for the Cultivation of Science, Kolkata, India

[3]National Brain Research Centre, Manesar, Haryana, India

[4] Innovative Life Discoveries Pvt. Ltd., Manesar, Haryana, India

Key Words: microRNAome, JEV, ceRNA, NOTCH

Address correspondence to

Vaccine and Infectious Disease Research Center (VIDRC)

Translational Health Science and Technology Institute (THSTI)

NCR Biotech Science Cluster, 3rd Milestone, Faridabad-Gurgaon Expressway, PO box #04, Faridabad -121001

e-mail: banerjeea@thsti.res.in.

MicroRNA Expression Profiling Using Affymetrix miRNA 3.0 array

RNA Isolation, Quality Control and Hybridization

Total RNA was isolated from the cell lines using RNeasy Mini kit according to the manufacturer's instructions (Qiagen, Hilden, Germany). Samples were collected from six-well plate in triplicate for each of the following time points: 6, 24 and 48 hours post infection (hpi) at MOI 5. Samples were frozen at −80°C for subsequent use in microarray experiments. RNA integrity number (RIN) was ascertained using Agilent 2100 Bioanalyzer (Agilent Technologies Inc, USA). Fragmentation, hybridization and scanning were performed according to the Affymetrix miRNA protocol, using the miRNA 3.0 array covering 5,339 probes for the human (Affymetrix, Santa Clara, CA, USA). After hybridization, washing and staining of the affymetrix miRNA 3.0 arrays was done and then scanning was done on Affymetrix scanner 7000 G. The raw data was extracted in the form of cel files and raw microarray data (CEL files) were preprocessed using RMA (Robust Multichip Average) algorithm in affymetrix expression console tool.

Differential miRNA Analysis

Statistical analysis was performed for the identification of differentially expressed miRNA (DEM) in infected and uninfected JEV groups by using GeneSpring GX 12.1. The One-way ANOVA method was applied for assessing the statistically significant DEM as well as consistence expressed miRNAs among three type of infected group. The *P*-value was calculated using asymptotic method and significance cutoff was set to 0.05. Then, these differentially expresses miRNAs probe were chosen to perform fold change analysis using moderated *t*-test (p<0.05) and probes which satisfied a fold change ≥1.5 between "infected 6 h & Uninfected 6 h, infected 24 h & Uninfected 24 h, infected 48 h & Uninfected 48 h was subjected for the cluster analysis, pathway analysis and target gene prediction.

mRNA array: Clustering Analysis

To determine the relationship between global miRNA or mRNA expressions for various time points, principal component analysis (PCA) was performed using all probe sets on the array. The data of ANOVA DEM including miRNA Ids and normalized expression values were imported into Genesis software for unsupervised hierarchical cluster analysis (HCL). The average linkage hierarchical clustering method was done on both miRNAs and samples. The heat map images were generated for the top expressed miRNAs in each three group (different time points: 6 h, 24 h, 48 h) of comparison using t-test & FC $\geq$1.5.

Supplementary Figure legend

Figure S1 JEV infection in CHME3 cells. (A) Cells were infected with infectious virus at a MOI of 5 or mock infected cell supernatant. At 6, 24 and 48 hpi cells were lysed and total RNA was extracted. qRT-PCR was done to detect JEV RNA using specific primers. Fold change of three set of samples were shown (left panel). Viral titer was calculated using supernatant collected at different time points by plaque assay. Data are represented as means ± SD (right panel). (B) JEV NS1 protein expression is depicted at 6, 24 and 48 hpi checked by western blot. GAPDH is used as loading control. (C) IF represents JEV E protein expression in CHME3 cells after 48 h pi. Green represents viral E protein and DAPI (blue) is used to stain the nucleus.

A

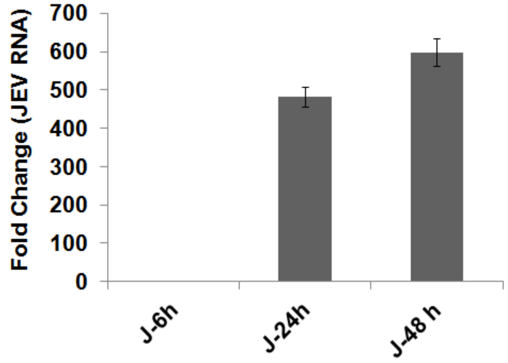

Exp set	Pfu/ml	Mean±SD
**J-24h**	6.00×10^{3}	$7.34\pm1.76\times10^{3}$
	6.67×10^{3}	
	9.34×10^{3}	
**J-48h**	1.43×10^{4}	$1.76\pm0.29\times10^{4}$
	2.00×10^{4}	
	1.87×10^{4}	

B

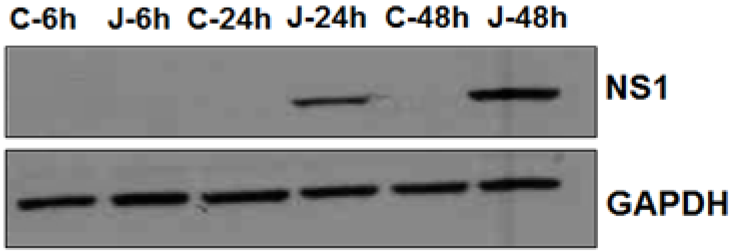

C

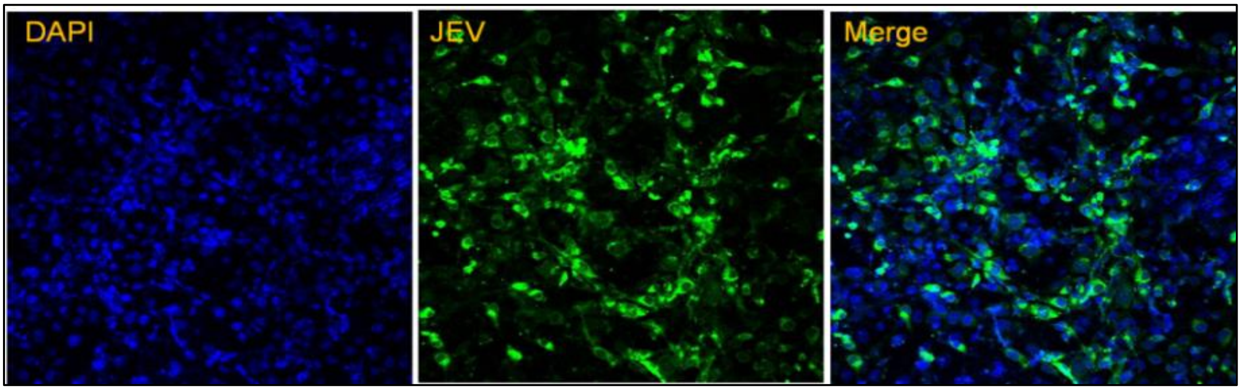

Fig S1

Table S1. List of Differentially expressed microRNAs

Signature miRNAs at 6 hpi			Signature miRNAs at 24 hpi
**Mirbase ID**	**Fold change (1.5, P<0.05)**	**Regulation**	**Mirbase ID**
hsa-mir-654	5.943	up	hsa-mir-4725
hsa-mir-1913	2.621	up	hsa-mir-4507
hsa-mir-4754	-2.219	down	hsa-mir-371b
hsa-mir-920	-2.558	down	hsa-mir-4505
hsa-mir-3132	-2.827	down	hsa-mir-572
hsa-mir-4445	-3.251	down	hsa-mir-2861
hsa-mir-198	-3.299	down	hsa-mir-4734
hsa-mir-4476	-3.32	down	hsa-mir-3185
hsa-mir-4314	-4.284	down	hsa-mir-4674
hsa-mir-4779	-4.426	down	hsa-mir-4466
hsa-mir-4423	-4.907	down	hsa-mir-4467
hsa-mir-184	-5.258	down	hsa-mir-4800
hsa-mir-4773	-5.443	down	hsa-mir-4508
hsa-mir-3128	-5.822	down	hsa-mir-4690
hsa-mir-3667	-6.212	down	hsa-mir-3656
hsa-mir-4468	-8.24	down	hsa-mir-3621
hsa-mir-4716	-9.091	down	hsa-mir-4492
hsa-mir-4753	-9.654	down	hsa-mir-1915
hsa-mir-4722	1.985	up	hsa-mir-638
hsa-mir-885-5p	1.65	up	hsa-mir-4785
hsa-mir-4763	1.564	up	hsa-let-7a-1
hsa-mir-572	1.556	up	hsa-mir-330
hsa-mir-129-5p	1.519	up	hsa-mir-103b-1
hsa-mir-4526	1.506	up	hsa-mir-3615
hsa-mir-101-1	1.578	up	hsa-mir-326
			hsa-mir-3127
			hsa-mir-29b-2
			hsa-mir-145
			hsa-mir-1304
			hsa-mir-130b
			hsa-mir-129-5p
			hsa-mir-1913
			hsa-mir-4722
			hsa-mir-4734
			hsa-mir-3687
			hsa-mir-4526
			hsa-mir-320e

Signature miRNAs at 48 hpi

Fold change (1.5, P<0.05)	Regulation	Mirbase ID	Fold change (1.5, P<0.05)
3.165	up	hsa-mir-4793	10.253
2.856	up	hsa-mir-3687	9.497
2.847	up	hsa-mir-5096	7.431
2.719	up	hsa-mir-4507	6.112
2.581	up	hsa-mir-4505	5.578
2.454	up	hsa-mir-572	5.578
2.38	up	hsa-mir-129-1	5.505
2.359	up	hsa-mir-1587	5.11
2.282	up	hsa-mir-4532	5.032
2.13	up	hsa-mir-1254	4.755
2.095	up	hsa-mir-4530	4.519
2.095	up	hsa-mir-675	4.23
2.095	up	hsa-mir-4440	4.229
2.062	up	hsa-mir-4417	4.201
2.02	up	hsa-mir-4651	4.037
2.013	up	hsa-mir-4484	3.884
2.002	up	hsa-mir-3648	3.819
1.991	up	hsa-mir-1909	3.814
1.98	up	hsa-mir-4508	3.752
1.93	up	hsa-mir-3188	3.74
-1.847	down	hsa-mir-1280	-1.752
-1.905	down	hsa-mir-409	-1.875
-1.994	down	hsa-mir-15a	-1.913
-2.022	down	hsa-mir-181a	-1.954
-2.201	down	hsa-let-7d	-2.765
-2.471	down	hsa-mir-145	-3.875
-2.478	down	hsa-mir-128	-1.715
-2.605	down	hsa-mir-130b	-3.71
-2.739	down	hsa-mir-129-3p	-2.11
-1.654	down	hsa-mir-21	-1.62
2.712	up	hsa-mir-181c	-1.81
1.608	up	hsa-mir-335	-4.34
1.798	up	hsa-mir-186	-2.38
2.517	up	hsa-mir-22	-1.53
3.341	up	hsa-miR-27b	-2.16
1.727	up	hsa-miR-320e	2.652
1.564	up	hsa-miR-4690	3.375
		hsa-miR-3148	4.34
		hsa-miR-4470	3.28
		hsa-miR-3180	1.759
		hsa-miR-4526	7.266
		hsa-miR-4695	3.016

Regulation
up
up
up
up
up
up
up
up
up
up
up
up
up
up
up
up
up
up
up
up
down
down
down
down
down
down
down
down
down
down
down
down
down
down
down
up
up
up
up
up
up
up

Table S2. MicroRNAs common to all three time points that are either up or down regulated during JEV infection

Mirbase ID	p	Fold Change (6 hpi)	Regulation	Fold Change (24 hpi)	Regulation	Fold Change (48 hpi)	Regulation
hsa-mir-197-3p	7.60E-05	-1.374	down	-1.491	down	-1.606	down
hsa-mir-320e	2.66E-05	1.218	up	1.564	up	2.652	up
hsa-mir-3648	4.55E-10	1.409	up	1.482	up	4.365	up
hsa-mir-3687	2.08E-08	1.119	up	3.341	up	11.686	up
hsa-mir-4508	2.34E-11	1.030	up	2.073	up	4.123	up
hsa-mir-4722	4.86E-05	1.985	up	1.798	up	2.225	up
hsa-mir-4734	8.14E-08	1.049	up	2.517	up	3.480	up
hsa-mir-4763	4.74E-04	1.564	up	1.187	up	4.201	up
hsa-mir-572	2.72E-09	1.5567	up	2.482	up	5.763	up
hsa-mir-129-5p	1.22E-06	1.519	up	2.712	up	7.143	up
hsa-mir-1913	1.14E-04	2.830	up	1.608	up	4.863	up
hsa-mir-4526	2.67E-07	1.506	up	1.727	up	7.266	up

Table S3. Brain Specific miRNAs modulated during JEV infection in microglia are also involved in neurological Diseases

Mirbase ID	p	Fold Change (6 hpi)	Regulation	Fold Change (24 hpi)	Regulation	Fold Change (48 hpi)	Regulation
hsa-mir-196a	1.76E-02	1.040	up	-1.168	down	-1.887	down
hsa-mir-128	3.30E-05	1.167	up	-1.0599	down	-1.715	down
hsa-mir-132	2.61E-04	-1.133	down	-1.394	down	-1.198	down
hsa-mir-29c-3p	3.39E-03	2.02	up	1.07	up	-2.48	down
hsa-mir-222	3.49E-03	-1.061	down	-1.066	down	-1.005	down
hsa-mir-9-3	1.28E-02	1.069	up	-1.016	down	-1.167	down
hsa-mir-7-1	1.17E-04	1.265	up	-1.369	down	-2.240	down
hsa-mir-101-1	4.06E-02	1.578	up	-1.459	down	-1.186	down
hsa-mir-155	3.32E-04	-1.074	down	1.288	up	1.494	up
hsa-mir-449b	2.24E-02	1.063	up	1.555	up	2.965	up
hsa-mir-130b	3.56E-02	1.895	up	-1.654	down	-3.710	down
hsa-mir-129	1.22E-06	1.519	up	2.712	up	7.143	up
hsa-mir-126	3.59E-02	1.155	up	-1.217	down	-1.456	down
hsa-let-7d	6.77E-04	1.497	up	1.194	up	-2.738	down
hsa-let-7e	2.97E-05	1.477	up	-1.857	down	-2.885	down
hsa-let-7f	1.13E-02	1.445	up	1.030	up	-1.972	down
hsa-let-7a-1	4.28E-03	1.413	up	-2.135	down	-1.090	down
hsa-let-7g	9.91E-03	1.247	up	-1.058	down	-1.791	down
hsa-let-7a-2	2.30E-06	1.122	up	-1.152	down	-2.774	down
hsa-let-7d	5.04E-03	1.012	up	-1.038	down	-1.369	down
hsa-let-7a	3.62E-02	1.007	up	-1.054	down	-1.123	down
hsa-let-7c	1.82E-02	-1.159	down	-1.060	down	-1.094	down

Table S4 Functional distribution of differentially expressed genes at 24 and 48 hpi.

Down regulated gene (%)		
**GO Term (GO ID)**	**24 hpi**	**48 hpi**
binding (GO:0005488)	66.26	75.8
protein binding (GO:0005515)	55.8	66.88
catalytic activity (GO:0003824)	23.3	24.8
nucleic acid binding (GO:0003676)	12.88	8.28
transferase activity (GO:0016740)	11.66	10.19
hydrolase activity (GO:0016787)	6.13	8.92
signal transducer activity (GO:0004871)	3.68	12.1
oxidoreductase activity (GO:0016491)	3.68	5.1
transporter activity (GO:0005215)	3.68	2.55
ligase activity (GO:0016874)	2.45	1.56
receptor activity (GO:0004872)	2.45	9.55
structural molecule activity (GO:0005198)	7.6	1.89
kinase activity (GO:0016301)	5.73	2
**UP regulated gene (%)**		
**GO Term (GO ID)**	**24 hpi**	**48 hpi**
ion binding (GO:0043167)	35.71	32.20
RNA binding (GO:0003723)	16.96	9.30
enzyme binding (GO:0019899)	16.07	6.12
DNA binding (GO:0003677)	13.39	14.29
nucleic acid binding transcription factor activity (GO:0001071)	10.71	8.57
signal transducer activity (GO:0004871)	8.93	6.94
enzyme regulator activity (GO:0030234)	7.14	1.29
ligase activity (GO:0016874)	6.25	2.45
protein binding transcription factor activity (GO:0000988)	6.25	1.59
transcription factor binding (GO:0008134)	5.36	2.86
kinase activity (GO:0016301)	3.57	2.45
cytoskeletal protein binding (GO:0008092)	3.57	2.04
GTPase activity (GO:0003924)	3.57	1.54
lipid binding (GO:0008289)	3.57	2.45
transmembrane transporter activity (GO:0022857)	3.57	2.45
helicase activity (GO:0004386)	2.68	1.56
mRNA binding (GO:0003729)	2.68	1.22

Table S5. List of highly enriched pathways modulated by differentially expressed miRNAs during JEV infection

Term	Count	P-Value	Fold Enrichment	Bonferroni	Benjamini	FDR
Neurotrophin signaling pathway	45	4.00E-08	2.3	7.20E-06	3.60E-06	4.90E-05
MAPK signaling pathway	77	4.40E-08	1.8	7.90E-06	2.60E-06	5.40E-05
Wnt signaling pathway	49	4.70E-07	2.1	8.40E-05	2.10E-05	5.70E-04
Axon guidance	43	1.20E-06	2.1	2.20E-04	3.60E-05	1.50E-03
Regulation of actin cytoskeleton	62	1.10E-06	1.8	2.10E-04	4.10E-05	1.40E-03
TGF-beta signaling pathway	32	3.80E-06	2.3	6.80E-04	7.60E-05	4.70E-03
Adherens junction	28	2.20E-05	2.3	4.00E-03	3.30E-04	2.70E-02
Focal adhesion	53	1.10E-04	1.7	2.00E-02	1.20E-03	1.40E-01
ErbB signaling pathway	27	6.10E-04	2	1.00E-01	5.40E-03	7.40E-01
Glioma	21	1.10E-03	2.1	1.80E-01	8.60E-03	1.40E+00
Fc gamma R-mediated phagocytosis	28	1.10E-03	1.9	1.80E-01	8.40E-03	1.40E+00
Phosphatidylinositol signaling system	23	1.70E-03	2	2.60E-01	1.20E-02	2.10E+00
Calcium signaling pathway	44	1.60E-03	1.6	2.40E-01	1.10E-02	1.90E+00
Cell adhesion molecules (CAMs)	32	1.20E-02	1.5	8.90E-01	6.10E-02	1.40E+01
Ubiquitin mediated proteolysis	33	1.20E-02	1.5	8.80E-01	6.10E-02	1.40E+01
GnRH signaling pathway	25	1.50E-02	1.6	9.40E-01	7.10E-02	1.70E+01
Gap junction	23	1.80E-02	1.6	9.60E-01	8.00E-02	2.00E+01
Notch signaling pathway	14	2.60E-02	1.9	9.90E-01	1.00E-01	2.80E+01
mTOR signaling pathway	15	2.70E-02	1.8	9.90E-01	1.00E-01	2.80E+01
Cell cycle	29	3.00E-02	1.5	1.00E+00	1.10E-01	3.10E+01

Table S6. List of predicted microRNAs and their target genes associated with multiple signaling pathway

Pathway	miRNA	Fold Change (48 hpi)	Target genes (P <0.05)	Fold Change (48 hpi)
NOTCH	miR-26b	-2.12	JAG1	3.23
	miR-34c	-2.42	NOTCH1	2.14
	miR-145	-4.37		
	miR-374b	-4.18	DLL1	2.51
Neurotrophin Signaling pathway	miR-3646	5.6	TNRC6B	-1.34
	miR-3148	4.34	CREB1	1.18
	Mir-129	4.11	MAPK1	1.21
	miR-4672	3.42	GSK3B	-1.37
	miR-4470	3.28	RAP1A	-1.45
	miR-3687	3.5	NCS1	-1.42
	miR-206	-1.32	NGFR	10.58
	miR-27a	-1.62	NGFR	10.58
	miR-27b	-2.16	NGFR	10.58
	miR-214	-1.42	NGFR	10.58
	miR-572	5.31	ADBRK1	-1.31
APOPTOSIS	miR-181c	-1.80	TNF	1.90
	miR-21	-1.62	FAS	5.23
	miR-210	-1.23	BCL2	5.12
	Let7e	-2.56	Caspase3	2.38
	miR-143	-1.97	Caspase 8	1.51
	miR-129-3p	-2.11	Caspase 8	1.79
	miR-98	-2.68	Caspase 8	1.79
	miR-335	-4.34	Caspase7	1.69
Inflammatory pathway	miR-146a	-1.30	CFH,	2.30
	miR-146a	-1.30	TRAF6	2.09
	miR-149	-3.15	TLR4	1.75
	miR-125b	-1.18	MyD88	2.51
	miR-132	-1.25	TNF	1.90
	miR-186	-2.38	ISG15	3.98
	miR-22	-1.53	IRF8	4.52
	miR-155	1.57	IRF8	4.52
	miR-155	1.57	TLR3	1.90
	miR-155	1.57	MAPK	-1.72
